# Bells Test: Are there differences in performance between adult groups aged 40-59 and 60-75?

**DOI:** 10.1590/1980-57642016dn11-010007

**Published:** 2017

**Authors:** Silvio Cesar Escovar Paiva, Vanisa Fante Viapiana, Caroline de Oliveira Cardoso, Rochele Paz Fonseca

**Affiliations:** 1 Universidade Federal do Rio Grande do Sul (UFRGS), Porto Alegre RS, Brazil.; 2 Pontifícia Universidade Católica do Rio Grande do Sul (PUCRS), Porto Alegre RS, Brazil.

**Keywords:** age, neuropsychological assessment, Bells Test, focused attention, visual hemineglect

## Abstract

**Objective:**

To verify whether differences exist between groups of Brazilian adults aged 40-59 and 60-75 in respective performance on the Bells Test, given the dearth of literature investigating the relationship between focused visual attention and the age factor.

**Methods:**

Eighty-four neurologically healthy adults (half aged 40-59 and half 60-75) with high educational level (40-59 years group: M=17.75 years' education; SD=4.00; 60-75 years group: M=15.85 years education; SD=3.19) were assessed using the Bells Test. Data on accuracy and processing speed were compared between groups by ANCOVA, controlled for the covariates education and frequency of reading and writing habits.

**Results:**

There were no significant differences between the age groups.

**Conclusion:**

It is suggested that aging influences sustained and focused attention and speed processing after 75 years of age on visual cancellation paradigms, when executive and attentional changes tend to be more marked. Further studies should investigate healthy older and oldest-old adults, as well as groups with low and intermediate educational backgrounds. In addition, Brazilian clinical populations should also be characterized, particularly those with neurological disorders that might have visual hemineglect.

## Abstract (pt)

**Objetivo:**

Verificar se existem diferenças entre grupos de adultos brasileiros de 40 a 59 e 60 a 75 anos de idade na performance no Teste dos Sinos, uma vez que há uma literatura reduzida que aborda as relações entre a atenção visual focalizada e o fator de idade.

**Métodos:**

Foram avaliados 80 adultos (metade deles com idade entre 40 e 59 e outra metade com idade entre 60 e 75) com alto nível educacional (40-59 anos de idade: M=17,75, DP=4,00; 60-75 anos de idade: M=15,85, DP=3,19) e saudáveis neurologicamente, por meio do Teste dos Sinos. Os dados de precisão e velocidade de processamento foram comparados entre os grupos por uma análise ANCOVA, controlando as covariáveis educação e frequência de hábitos de leitura e escrita.

**Resultados:**

Não houve diferenças significativas entre os grupos etários.

**Conclusão:**

Sugere-se que a influência do envelhecimento na atenção sustentada e focalizada e velocidade de processamento após os 75 anos de idade em paradigmas de cancelamento visual, quando as mudanças executivas e atencionais tendem a ser mais notáveis. Estudos futuros devem investigar adultos saudáveis mais velhos e longevos, bem como, adultos com níveis educacionais baixos e intermediários. Ademais, populações clínicas brasileiras também devem ser caracterizadas, principalmente com transtornos neurológicos que podem apresentar heminegligência visual.

## INTRODUCTION

Consistent with data regarding the Brazilian elderly population from the Brazilian Institute of Geography and Statistics – "Instituto Brasileiro de Georgrafia e Estatística" – IBGE,^1^ the Brazilian population is aging, with almost 23 million people over 60 years of age in the country. As this transition is in fact a worldwide phenomenon, studies in the area of developmental neuropsychology have investigated the aging process and its subsequent changes in cognitive domains such as memory, executive functions, language and attention in order to better understand the processing of working memory,^2–4^ processing speed and problem-solving,^5,6^ oral and written linguistic abilities,^7,8^ as well as visual spatial attention.^9,10^ Neuropsychological assessment is one of the tools employed to investigate the biopsychosocial process of aging, accomplished through observation and clinical interviews together with the administration of standardized instruments evaluating different cognitive processes, both quantitatively and qualitatively. Neuropsychological assessment studies have increasingly focused on the relationships of sociocultural and biological variables with cognitive processing. Herrera-Guzmán, Peña-Casanova, Lara, Gudayol-Ferré, & Böhm^11^ maintain that the sociodemographic aspects that might influence performance on a cognitive instrument should be known so that clinicians and/or researchers may differentiate the effect of the neurological or psychiatric status from aspects such as age, sex and education,^12^ among others. Both low educational attainment and older age can decrease scores on neuropsychological tests, leading to false-positive results. In conjunction with these aspects, education^13^ and socioeconomic status^14^ are the sociocultural variables most investigated in healthy adults. Additionally, biological variables such as age^15,16^ and sex^17^ are important.

With regard to age, cross-sectional comparison of extreme groups is the most frequently used design, with studies focused on groups of young adults *versus* older adults.^18,19^ In relation to this bias in the literature, some studies point to a decline in cognitive functions,^20^ while cross-sectional and longitudinal studies suggest that cognitive decline starts from adult age.^21^ Supporting this framework, some cross-sectional studies in the international literature suggest that older adults might have poorer performance regarding speed and accuracy when compared to young adults.^22^ However, some studies in the national (Brazilian sample) and international milieu indicate that healthy older adults can maintain steady accuracy performance befitting normal aging.^23-25^

Indisputably there are cognitive changes in the process of growing older that are age related, although this can involve different levels and functions. Furthermore, Ska & Joanette^26^ suggested the notion that neurobiological and cognitive changes might lead the elderly to perform worse, better, or as well as, younger adults, depending on the cognitive function.

Although there is a consensus in the literature on cognitive changes in healthy older adults, the same is not true regarding the onset of this cognitive decline.^21^ In this context, three theories exist in the developmental neuropsychology approach to cognitive decline in aging. Some authors advocate that, despite the cognitive changes promoted by normal aging, there should be, in contrast, a large repertoire of new strategies to overcome difficulties in other functions.^27,28^ Other authors, in turn, argue that there is inconsistent decline in most tasks during aging, since some skills decline more than others.^29^ Salthouse^30^ suggested that one of the origins of cognitive impairment is a deficit in speed processing. Hence, the author identified that performance on tasks involving speed or measuring response time show striking differences with regard to age. On the other hand, other researchers state that relevant cognitive changes only occur from the age of 75 years onwards.^31^ Recent intraindividual studies show results indicating that developmental changes increase with age, affecting memory, reaction time and sensory motor tasks.^29^

Thus, the importance of studying and understanding the influence of the biological and demographic variables on performance of cognitive tasks in gleaning a better understanding of the possibilities of developmental pathways is clear. Despite the relevance of these issues, few studies have investigated the differences among ages in adulthood, for instance, comparing middle-aged populations with the oldest-old.^32-34^

Cancellation paradigms are often used for neuropsychological assessment of attention in aging.^35-37^ A well-known cancellation instrument for assessing hemineglect syndrome is the Bells Test, developed by Gauthier, Dehaut, & Joanette.^38^ It consists of cancelling the target pictures (Bells) after mixing them among distracters in a pseudo randomized way so as to investigate attentional functions, visual perception and processing speed. In the literature, the Bells Test is considered a sensitive test for diagnosing visual hemineglect.^39-41^ Brazilian studies have been conducted to investigate the best form of applying the test.^42^ Other studies have used different versions of the Bells Test to assess children with Attention Deficit Hyperactivity Disorder (ADHD) along with a control group,^43^ as well as to investigate how differences in educational level impact performance of young adults.^44^

However, few studies have examined samples of older adults. An international study used the Bells Test to compare four age groups, including middle-aged adults and older adults, reporting lower performance of the latter in accuracy and performance time.^45^ These data are consistent with other studies, with reference to processing speed in cancellation tests (for example^37,46^). The aim of this study was to investigate whether differences exist between adult groups aged 40 to 59, representing middle-aged adults, and individuals aged 60 to 75, representing older adults, in performance on the Bells Test. The hypotheses of this study are:

1. there is no difference in accuracy between age groups;
2. healthy older adults need more time to perform the cancellation test than healthy middle-aged adults.

## METHODS

**Participants.** Eighty neurologically healthy adults aged 40 to 75, with at least 12 years of formal education, volunteered for this study; participants were split into two age groups:

(1) n=40 middle-aged adults, 40 to 59 years, and
(2) n=40 older adults, 60 to 75 years.

Convenience sampling was employed to select individuals in universities and business environments as well as community centers. Only native Brazilians, speakers of Brazilian Portuguese, with 12 or more years of formal education and without current or prior history of neurological or psychiatric self-reported diseases were included in the sample. None of the participants exhibited absence of uncorrected visual or auditory sensory disturbances (assessed by self-report using a socio-cultural and health aspects questionnaire) or signs suggesting depression (Beck Depression Inventory – BDI-II, by Beck, Steer, & Brown,^47^ adapted for the Brazilian population by Gorenstein, Andrade, Vieira Filho, Tung, & Artes^48^ with score ≥19 points); psychiatric disorders (screened by *Self Report Questionnaire* – SRQ, translation by Mari & Williams,^49^ adopting cut-offs of 5/6 for men and 7/8 for women); dementia (score ≥24 points on the Mini-Mental State Examination MMSE, by Folstein, Folstein, & McHugh^50^ and Brucki, Nitrini, Caramelli. Bertolucci, & Okamoto;^51^ history of alcoholism (CAGE screening^52^ Brazilian version used in Amaral & Malbergier;^53^ current or previous abuse of illegal drugs, benzodiazepines, neuroleptics or antipsychotics in the past six months (assessed by self-report using a socio-cultural and health aspects questionnaire). Besides these criteria, participants were included only if they attained a score ≥7 on the vocabulary and blocks subtests of the WAIS-III.^54^ Accordingly, Table 1 displays descriptive data on socio-demographic and clinical characteristics of the sample. Regarding the frequency of reading and writing habits, these were assigned scores from 4 (every day) to 0 (never), where the total score of frequency of reading and writing habits was the sum of the partial reading of magazines, newspapers, books, and others, including emails (maximum score of 16), and writing of texts, messages, and others, such as emails (12 points).

**Table 1:** Characteristics of the sample according to sociodemographic and clinical data by group.

Variables characterizing sample	Groups						
	Adults 40-59 (n=40)			Adults 60-75 (n=40)			
	M	SD		M	SD	t	p
Age (years)	49.33	4.948		66.35	3.971	16.971	≤0.001
Education (years)	17.75	4.005		15.85	3.191	2.347	0.021
Frequency of reading and writing habits	18.13	4.316		14.98	4.583	3.165	0.002
Sex distribution (F)	29 (72. 5%)			27 (67. 5%)			
Sex distribution (M)	11 (27. 5%)			13 (32. 5%)		-	0.808
MMSE Score	28.65	1.718		27.88	1.977	1.872	0.065
BDI-II Score	5.23	4.666		7.03	4.610	-1.736	0.087
Vocab weighted score WAIS-III	11.19	2.088		10.77	1.784	0.916	0.363
Blocks weighted score WAIS-III	13.68	2.329		13.56	2.269	0.205	0.838

M=mean; SD=standard deviation; MMSE=Mini-Mental State Examination; BDI-II=Beck Depression Inventory; WAIS-III=Wechsler Intelligence Scale for Adults; Vocab=vocabulary; p value=based on Student's t-Test for independent samples; with df=78 for all variables.

The sample characteristics given in Table 1 reveal significant differences between groups in relation to education background and frequency of reading and writing habits. With regard to sex, there were no significant differences in gender distribution between groups (χ^2^(1)=0.238) and no differences were observed for clinical variables.

**Procedures and instrument.** Participants were assessed individually in an appropriate environment, respecting all ethical standards in research with humans. All subjects provided informed consent, after approval of the study by the Research Ethics Committee of PUCRS. Participants were assessed individually by trained a professional in a session lasting an average of one hour with application of the Bells Test, Sociocultural and health questionnaire,^55,56^ the reading and writing inventory^57^ and the instruments for characterizing the sample and inclusion criteria assessment.

**Instrument.** The Bells Test, originally called the *Test des Cloches*,^38^ is an instrument entailing cancellation of targets (bells) among distracters, which assesses visual selective and focused attention, visual perception and visual motor processing speed, adapted to Brazilian Portuguese.^58^ In this test, the examinee must cancel 35 pseudo randomized bells found on a horizontal sheet of paper mixed with another 315 figures. Therefore, the individual's task is to locate the bells and cross them out in the shortest possible time. The bells are located in seven columns, three in the right visual field, three on the left, and one in the center, leading to an analysis on whether the omissions could be associated with a specific region of the two visual fields. The main scores are generated from a quantitative interpretation, number of omissions and performance time in the first and second applications, and a qualitative interpretation, record of the column where the first bell was cancelled and identification of the bell search and cancellation strategy. There are two time records in the Brazilian version: time 1, before the systematic instruction to review whether all the bells have been cancelled, and time 2, after this instruction.^58^

The Sociocultural and health questionnaire^55,56^ includes questions about gender, age, education, socioeconomic status, frequency of reading and writing, and handedness. It also allows for the identification of health conditions which constitute exclusion criteria. The reading and writing inventory^57^ inquires as to the frequency with which individuals read newspapers, magazines, books or other types of material, and write essays, notes or other types of text. The frequency of each activity is assigned a score from 0 to 4 depending on whether the individual engages in the activity every day (4), some days a week (3), once a week (1), or never (0), for a maximum possible score of 16 for reading and 12 for writing habits. The frequency of these activities is classified as high or low depending on whether the summed scores of reading and writing frequency falls above or below 14.

**Data analysis.** The comparison between groups regarding performance measured on the Bells Test was analyzed using the ANCOVA statistical test, defining years' education and frequency of reading and writing habits as covariates. The sample distribution of the contrasting groups according to the column of the first bell cancellation and search and cancellation strategies were analyzed using the Chi-square test. The software SPSS, version 15.0 was used for statistical analyses. The significance level was p≤0.05.

## RESULTS

Table 2 shows the average standard deviation of scores on the Bells Test in groups aged 40 to 59 years old and 60 to 75 years old.

**Table 2:** Mean scores, standard deviation and Bells Test score significance level by group.

Bells scores	Groups					p value age	p value education	p value freq. hab.	F
	40-59 years			60-75 years					
	M	SD		M	SD				
Total omissions in time 1	1.30	1.588		1.88	2.564	0.423	0.651	0.585	4.914
Total omissions in time 2	0.45	0.846		0.45	0.815	0.650	0.391	0.461	<0.001
Total time 1	108.22	39.29		127.44	52.27	0.151	0.733	0.312	3.985
Total time 2	55.43	19.77		63.14	29.90	0.489	0.423	0.293	3.249

Note: freq. hab.=reading and writing inventory.57 For all scores, df=78.

According to data shown in Table 2, there are no significant differences between the groups as regards the variables accuracy and time performance on the Bells Test. Despite controlling for the possible effects of the covariates, the cognitive performance measured may not have been influenced directly by years of education or frequency of reading habits.

Concerning the position frequency of the first bell cancelled, the groups showed no differences in this distribution (p=0.622). Most participants chose the first column when cancelling the first bell in both age groups (75% adults aged 40-59 years and 77.5% adults 60-75 years). Therefore, in this study, the visual search strategy in general began on the left. Table 3 shows the distribution of groups according to the approach used for cancellation: mixed horizontal (left to right or right to left), mixed vertical (top to bottom or bottom to top), mixed, and unorganized crossing.

**Table 3:** Frequency of cancellation strategies adopted by participants in each group.

Strategies	Groups			
	40-59 years		60-75 years	
Mixed horizontal	10	25.0%	12	30.0%
Mixed vertical	19	47.5%	16	40.0%
Mixed	2	5.0%	6	15.0%
Unorganized crossing	5	12.5%	4	10.0%

According to Table 3, regarding the qualitative performance of the cancellation strategy, there seems to be no difference between groups in the distribution of this variable. For a better understanding of the possible differences, the organized strategies were compared with the disorganized ones, revealing no significant difference in distribution between groups (χ^2^ (0.196)=1, p=0.658). Notably, the most common strategy used by both groups was the mixed vertical, followed by horizontal mixed.

## DISCUSSION

This comparative study of performance on the Bells Test among middle-aged adults and older adults showed no significant differences, neither in the quantitative analysis of accuracy and execution time or in the qualitative analysis of the column of cancellation of first bell, nor related to the search strategy of the target. In general, the data differed from most previous studies comparing middle-aged adults and elderly adults, which generally suggested that increasing age was associated with slower performance.^45^ With regard to accuracy, the results are conflicting, with some evidence of greater omissions for elderly adults,^45^ as well as the absence of differences compared to younger groups^37,46^ in cancellation tests. Thus, the initial hypothesis of this study on the absence of accuracy difference for age was confirmed, but the hypothesis of the presence of difference in processing speed was not.

The absence of significant differences among groups can be understood in light of three hypotheses to be discussed:

(1) as this instrument was originally devised for neurological patients, it may have been performed easily by the sample of older adults;
(2) given that all individuals were highly educated with high frequency of reading and writing habits, the elderly adults may have maintained adequate processing speed by having sufficient cognitive reserve;^60^
(3) since the age limit of elderly participants in this study was 75 years, differences in performance between older groups may occur when comparing adults aged 76 years or older; and
(4) The small sample size, where larger samples may lead to different results.

Regarding the first hypothesis, in the international literature, the Bells Test is one of the most renowned and frequently used tests to evaluate visual hemineglect, precisely for being suitable for the clinical examination of this neurological status. In studies comparing adults with unilateral brain damage and healthy controls, the clinical group had worse performance in accuracy.^39^ A study investigating stroke patients showed that patients with right brain damage had more omissions and also took longer to complete the Bells test than those with left brain damage and healthy controls.^41^

A study by Rousseaux et al.^45^ involving a healthy sample aged 20 to 80 in a randomized comparison with the Bells Test suggested that the number of omissions and performance time were influenced by age. Individuals aged between 65 and 79 years had poorer performance compared to the different groups. Other studies examining middle-aged adults and older adults on cancellation tests showed that the performance of elderly people was lower for execution time, but with no difference between groups in terms of accuracy found.^37,46^ Cardoso et al.^42^ suggested that older adults had greater psychomotor slowness when compared with young individuals and therefore may have impaired attention on the neuropsychological assessment. However, only a few studies have interpreted the effect of age on both accuracy and processing speed.^46^

Regarding the performance on the Bells Test, there are two ways to evaluate the individual qualitatively:

(1) search strategy; and
(2) column of first bell cancellation.

Data from this study on the evaluation of search strategy demonstrated that the results are consistent with previous studies indicating that healthy individuals have a scan strategy organized in systematic patterns: vertical and horizontal.^46^ Regarding the column of first bell cancellation, there is agreement between the our data and the study of Rousseaux et al.,^45^ which revealed that individuals began by circling a bell located in the left columns, with no influence of age or education level. These findings reinforce the idea that the Bells Test is relatively easy for neurologically healthy individuals and that performance time can be easily offset if the individual uses effective cognitive strategies. A Brazilian study investigated the effects of education on Bell test performance failed to find differences in the scores of total omissions and task execution time among individuals with high and low educational level but the authors found differences in qualitative scores. Subjects with high educational level tended to mark the first bell in column 1 and had a more organized visual search.^44^ Therefore, we considered that the relationship between education and cognitive performance can be explained by our second hypothesis. An educational process results in cognitive stimulation and can generate a cognitive reserve that might be protective against pathological aging.^60^ Education background has been identified as crucial in neuropsychological performance on tasks assessing several different functions such as attention.^61^ According to Stern (2002, 2009),^59,62^ this cognitive reserve may have a protective role against injury and brain dysfunction. Many factors contribute to the formation of a cognitive reserve, for instance, education, intellectual capacity and the type of work that people do throughout their lives. According to this theory, when an individual is exposed to a challenging task, it creates a series of connections between neurons and neuronal stimulation that causes them to form, over time, can result in a good cognitive reserve.

While studying cognitive reserve and aging^63,64^ it was found that the first acts as a protective factor against cognitive decline associated with age in healthy subjects. Additionally, there is further evidence that the healthy older adult population tends to have a higher educational background and also remains active and socially engaged.^11,12,65^ A Brazilian study showed that in developing countries neuropsychological performance of older adults is more influenced by socioeconomic than biological factors.^66^

The results found in this study are consistent with previous investigations indicating that high educational levels and frequent reading and writing habits are good predictors of performance on neuropsychological tests.^67,68^

Studies that investigated cognitive reserve and processing speed results have not identified any direct correlation between these two variables.^69^ In the present study, the sample may have offset processing speed, often reported in the literature as being reduced when compared to younger individuals. Furthermore, most studies reporting reduction in procedural speed have compared older adults to young adults, i.e., an extreme age group.^19^ The fact that our sample was composed of two consecutive age groups, middle-aged and older adults, and that there were no notable differences in processing speed on the cancellation of target bells may lead to the hypothesis that either the adults aged 40 to 59 years have already begun to exhibit longer execution time on cognitive tasks or that adults aged 75 years have not experienced a decline in processing speed in performing easier tasks.

As a complement to this reflection, a third possible explanation for the findings of this study is that differences between middle-aged adults and the older adults may have been found if an older group, aged 75 years or older were included. No differences in outcomes between middle-aged and elderly adults were found in the investigation of processing speed. These results are not consistent with studies evaluating the effects of age on performance in processing speed in general. According to Salthouse,^21^ cognitive decline is characterized by slow processing with age, which can be seen in simple tasks with pencil and paper which seek quick judgments of similarities and differences. This approach is congruent with the results found in the studies by Rousseaux et al.,^45^ and Uttl & Pilkenton-Taylor^37^ and Warren et al..^46^ Although there have been many reports in the last century of age-related differences in cognitive functioning, there is still controversy about the age of onset of cognitive decline. This lack of consensus is concerning because it emphasizes the issue, highlighting its importance both for practical and theoretical reasons, so as to enable the effective distinction of a healthy performance from a pathological one. Lowe & Reynolds^70^ underscore the importance of the inclusion of control samples consisting of young and middle-aged adults, besides the elderly, in order to show increase, sparing or possible decline in cognitive performance with aging. Although many studies have investigated the relationship between the variable age and cognitive functions, most have compared extreme age groups: young adults versus older adults.^19,22^ In research of processing speed in older adults, these subjects had poorer performance compared to younger elderly.^45^ Zibetti et al.^16^ investigated the effect of age on the processing of neuropsychological functions and found that, by controlling the variables education and frequency of reading and writing habits, participants older than 60 years showed one performance pattern for some functions while subjects aged 76 or older showed another. In studies of developmental neuropsychology, cognitive changes occur in dissociation with of aging.^26^

On the other hand, results in longitudinal studies suggest that most of the elderly population had no cognitive decline, i.e., a stable and benign evolutionary trajectory.^23,71^ However, the results of cross-sectional and longitudinal studies may be similar when comparable measures are used in both types of studies. Additionally, a systematic review study found that in developing countries the prevalence of dementia doubles for every five-year increase in age, ranging from 3% at 70 years of age to 20-30% at 85 years of age. Nevertheless, the authors concluded that in Brazil the overall prevalence of dementia among elderly could not be estimated because of the large social disparities.^71^

Although the current study contributed to better understand the effect of age groups on the performance of an attentional, speed processing and motor abilities-dependent task, it has some limitations. It is necessary to consider the sample size, as well as the sample age range. Since life expectancy has significantly increased in recent years, studies should include older populations (80-90 years old) in order to better investigate cognitive decline mechanisms. Towards clearer evidence of cut-off points for cognitive decline, further studies including participants representing all phases throughout adulthood are necessary. The findings of such studies should be carefully analyzed, assuming the inherent limitations of a cross-sectional design, which takes age as a group variable instead of a continuous factor.

It is important to conduct further investigation on the role of age in the processing examined by the Bells Test with older adults of different age groups and education background in order to better understand the results and establish benchmarks for clinical neuropsychology. Thus, for future studies, we suggest broadening the sample to include older-old adults, young and middle-aged adults and younger elderly, as well as participants with low and intermediate education background. After obtaining parameters of standard performance in Brazilian healthy samples, data from neurological clinical population that can present visual hemineglect syndrome should be considered. Finally, it might be useful to develop a version of the Bells Test with a higher degree of difficulty for the diagnosis of attention deficits and processing speed in populations that are healthy or have more subtle symptoms. Since this instrument was originally designed to assess populations with neuropsychological impairments, a more difficult version could discriminate milder perceptual-attention deficits, replicating its known sensitivity for hemineglect symptoms for other neuropsychiatric disorders, such as attention deficit hyperactivity disorder.

## References

[1] Brasil, IBGE Instituto Brasileiro de geografia e Estatística. Censo
Demográfico 2010.

[2] Beigneux K, Plaie T, Isingrini M (2007). Aging Effect on Visual and Spatial Components of Working
Memory. Int J Aging Hum Dev.

[3] Cabeza R (2002). Hemispheric asymmetry reduction in older adults: The HAROLD
model. Psychol Aging.

[4] Fama R, Sullivan E V (2015). Thalamic structures and associated cognitive functions: Relations
with age and aging. Neurosci Biobehav Rev.

[5] Feld JE, Sommers MS (2009). Lipreading, processing speed and Working Memory in Younger and
Older Adults. J Speech Lang Hear Res.

[6] Crawford S, Channon S (2002). Dissociation between performance on abstract tests of executive
function and problem solving in real-life-type situations in normal
aging. Aging Ment Health.

[7] Labos E, Del M, Zabala K (2009). Perfil de desempeño lingüístico en el adulto
mayor.

[8] Snitz BE, Unverzagt FW, Chang C-CH, Bilt J Vander, Gao S, Saxton J (2009). Effects of age, gender, education and race on two tests of
language ability in community-based older adults. Int Psychogeriatrics.

[9] Curran T, Hills A, Patterson MB, Strauss ME (2001). Effects of aging on visuospatial attention: an ERP
study. Neuropsychologia.

[10] Nagamatsu LS, Munkacsy M, Liu-Ambrose T, Handy TC (2013). Altered visual-spatial attention to task-irrelevant information
is associated with falls risk in older adults. Neuropsychologia.

[11] Herrera-Guzmán I, Peña-Casanova J, Lara JP, Gudayol-Ferré E, Böhm P (2004). Influence of age, sex, and education on the Visual Object and
Space Perception Battery (VOSP) in a healthy normal elderly
population. Clin Neuropsychol.

[12] Ihle A, Oris M, Fagot D, Baeriswyl M, Guichard E, Kliegel M (2015). The Association of Leisure Activities in Middle Adulthood with
Cognitive Performance in Old Age: The Moderating Role of Educational
Level. Gerontology.

[13] Gómez-Pérez E, Ostrosky-Solís F (2006). Attention and Memory Evaluation Across the Life Span:
Heterogeneous Effects of Age and Education. J Clin Exp Neuropsychol.

[14] Jang S-N, Choi Y-J, Kim D-H (2009). Association of socioeconomic status with successful ageing:
differences in the components of successful ageing. J Biosoc Sci.

[15] Ostrosky-Solís F, Gómez-Pérez ME, Matute E, Rosselli M, Ardila A, Pineda D (2007). Neuropsi Attention and Memory: a neuropsychological test battery
in Spanish with norms by age and educational level. Appl Neuropsychol.

[16] Zibetti MR, Gindri G, Pawlowski J, Fumagalli J, Alice M, Parente MP (2010). Estudo comparativo de funções
neuropsicológicas entre grupos etários de 21 a 90
anos. Rev Neuropsicol Latinoam.

[17] Varnava A, Halligan PW (2007). Influence of Age and Sex on Line Bisection: A Study of Normal
Performance with Implications for Visuospatial Neglect. Aging Neuropsychol Cogn.

[18] Geldmacher DS, Riedel TM (1999). Age effects on random-array letter cancellation
tests. Cogn Behav Neurol.

[19] Larson MJ, Clayson PE, Keith CM, Hunt IJ, Hedges DW, Nielsen BL (2016). Cognitive control adjustments in healthy older and younger
adults: Conflict adaptation, the error-related negativity (ERN), and
evidence of generalized decline with age. Biol Psychol.

[20] Salthouse TA (2004). What and When of Cognitive Aging. Curr Dir Psychol Sci.

[21] Salthouse TA (2009). When does age-related cognitive decline begin?
Neurobiol. Aging.

[22] Endrass T, Schreiber M, Kathmann N (2012). Speeding up older adults: Age-effects on error processing in
speed and accuracy conditions. Biol Psychol.

[23] Argimon II de L, Stein LM (2005). Habilidades cognitivas em indivíduos muito idosos: um
estudo longitudinal. Cad Saude Publica.

[24] Lucci G, Berchicci M, Spinelli D, Taddei F, Russo F Di (2013). The Effects of Aging on Conflict Detection. PLoS One.

[25] Ska B, Joanette Y (2006). Normal aging and cognition. Médecine Sci.

[26] Aine CJ, Woodruff CC, Knoefel JE, Adair JC, Hudson D, Qualls C (2006). Aging: Compensation or maturation?. Neuroimage.

[27] Parente MAMP, Wagner GP (2006). Teorias abrangentes sobre envelhecimento
cognitivo. Cogn Envelhec.

[28] Christensen H (2001). What cognitive changes can be expected with normal
ageing?. Aust N Z J Psychiatry.

[29] Salthouse TA (1996). The processing-speed theory of adult age differences in
cognition. Psychol Rev.

[30] Beni R De, Palladino P (2004). Decline in working memory updating through ageing: Intrusion
error analyses. Memory.

[31] Grady CL, Springer M V, Hongwanishkul D, McIntosh AR, Winocur G (2006). Age-related Changes in Brain Activity across the Adult
Lifespan. J Cogn Neurosci.

[32] LeBlanc J, Guise E de, Gosselin N, Feyz M (2006). Comparison of functional outcome following acute care in young,
middle-aged and elderly patients with traumatic brain injury. Brain Inj.

[33] Peña-Casanova J, Quinones-Ubeda S, Quintana-Aparicio M, Aguilar M, Badenes D, Molinuevo JL (2009). Spanish Multicenter Normative Studies (NEURONORMA Project): Norms
for Verbal Span, Visuospatial Span, Letter and Number Sequencing, Trail
Making Test, and Symbol Digit Modalities Test. Arch Clin Neuropsychol.

[34] Lezak MD (2004). Neuropsychological Assessment.

[35] Strauss E, Sherman EM, Spreen O (2006). A compendium of neuropsychological tests: Administration, norms,
and commentary. American Chemical Society.

[36] Uttl B, Pilkenton-Taylor C (2001). Letter Cancellation Performance Across the Adult Life
Span. Clin Neuropsychol.

[37] Gauthier L, Dehaut F, Joanette Y (1989). A quantitative and qualitative test for visual
neglect. Int J Clin Neuropsychol.

[38] Azouvi P, Samuel C, Louis-Dreyfus A, Bernati T, Bartolomeo P, Beis J-M (2002). Sensitivity of clinical and behavioural tests of spatial neglect
after right hemisphere stroke. J Neurol Neurosurg Psychiatry.

[39] Calvette LDF, Joanette Y, Fonseca RP (2013). Traumatismo cranioencencefálico: da ocorrência de
heminegligência e de déficit atencional por tarefas de
cancelamento. Av Psicol Latinoam.

[40] Oliveira CR, Calvette  L de F, Pagliarin KC, Fonseca RP (2016). Use of Bells Test in the Evaluation of the Hemineglect Post
Unilateral Stroke. J Neurol Neurosci.

[41] Cardoso CO, Silva RFC, Fonseca RP (2011). Teste de Cancelamento dos Sinos: comparação entre
duas versões. Gerais, Rev Interinst Psicol.

[42] Gonçalves HA, Mohr RM, Moraes AL, Siqueira L de S, Prando ML, Fonseca RP (2013). Componentes atencionais e de funções executivas em
meninos com TDAH: Dados de uma bateria neuropsicológica
flexível. J Bras Psiquiatr.

[43] Silva RFC da, Cardoso C de O, Fonseca RP (2012). Diferenças quanto à escolaridade em adultos no
desempenho no teste de cancelamento dos sinos. Estud Psicol.

[44] Rousseaux M, Beis JM, Martin Y, Bartolomeo P, Bernati T, Chokron S (2001). Présentation d'une batterie de dépistage de la
négligence spatiale. Rev Neurol. (Paris).

[45] Warren M, Moore JM, Vogtle LK (2008). Search Performance of Healthy Adults on Cancellation
Tests. Am J Occup Ther.

[46] Beck AT, Steer RA, Brown GK (1996). Beck depression inventory-II.

[47] Gorenstein C, Andrade L, Helio A, Vieira G, Tung TC, Artes R (1999). Psychometric Properties of the Portuguese Version of the Beck
Depression Inventory on Brazilian College Students. J Clin Psychol.

[48] Mari JJ, Williams P (1986). A validity study of a psychiatric screening questionnaire
(SRQ-20) in primary care in the city of Sao Paulo. Br J Psychiatry.

[49] Folstein MF, Folstein SE, McHugh PR (1975). "Mini-mental state": a practical method for grading the cognitive
state of patients for the clinician. J Psychiatr Res.

[50] Brucki SMD, Nitrini R, Caramelli P, Bertolucci PHF, Okamoto IH (2003). Sugestões para o uso do mini-exame do estado mental no
Brasil. Arq Neuropsiquiatr.

[51] Ewing J, Rouse BA (1970). Identifying the hidden alcoholic.

[52] Amaral RA do, Malbergier A (2004). Avaliação de instrumento de detecção
de problemas relacionados ao uso do álcool (CAGE) entre trabalhadores
da prefeitura do campus da Universidade de São Paulo (USP) - campus
capital. Rev Bras Psiquiatr.

[53] Wechsler D (1997). WAIS-III: Administration and scoring manual.

[54] Fonseca RP, Zimmermann N, Cotrena C, Cardoso C, Haag C, Grassi-Oliveira R (2012). Neuropsychological assessment of executive functions in traumatic
brain injury: hot and cold components. Psychol Neurosci.

[55] Fonseca RP, Zimmermann N, Pawlowski J, Oliveira CR, Gindri G, Scherer LC, J L-F, S. FS (2012). Métodos em avaliação
neuropsicológica: pressupostos gerais, neurocognitivos,
neuropsicolinguísticos e psicométricos no uso e
desenvolvimento de instrumentos. Métodos de Pesquisa em Neurociência Clínica e
Experimental.

[56] Pawlowski J, Remor E, Parente MA de MP, Salles JF de, Fonseca RP, Bandeira DR (2012). The influence of reading and writing habits associated with
education on the neuropsychological performance of Brazilian
adults. Read Writ.

[57] Fonseca RP, Parente MAM, Ortiz KZ, Soares ECS, Scherer LC, Gauthier L Teste de Cancelamento dos Sinos.

[58] Stern Y (2009). Cognitive reserve. Neuropsychologia.

[59] Kramer AF, Colcombe SJ, McAuley E, Scalf PE, Erickson KI (2005). Fitness, aging and neurocognitive function. Neurobiol Aging.

[60] Fonseca RP, Zimmermann N, Scherer LC, Parente MA de MP, Ska B (2010). Episodic memory, concentrated attention and processing speed in
aging: A comparative study of Brazilian age groups. Dement Neuropsychol.

[61] Stern Y (2002). What is cognitive reserve? Theory and research application of the
reserve concept. J Int Neuropsychol Soc.

[62] Corral M, Rodriguez M, Amenedo E, Sanchez JL, Diaz F (2006). Cognitive Reserve, Age and Neuropsychological Performance in
Healthy Participants. Dev Neuropsychol.

[63] Álvarez MR, Rodríguez JLS (2004). Reserva cognitiva y demencia. An Psicol.

[64] Newson RS, Kemps EB (2005). General Lifestyle Activities as a Predictor of Current Cognition
and Cognitive Change in Older Adults: A Cross-Sectional and Longitudinal
Examination. J Gerontol B Psychol Sci Soc Sci.

[65] Chaves ML, Camozzato AL, Eizirik CL, Kaye J (2009). Predictors of Normal and Successful Aging Among Urban-Dwelling
Elderly Brazilians. J Gerontol Psychol Sci.

[66] Byrd DA, Touradji PEGAH, Tang MX, Manly JJ (2004). Cancellation test performance in African American, Hispanic and
White elderly. J Int Neuropsychol Soc.

[67] Ostrosky-Solís F, Ardila A, Rosselli M (1999). NEUROPSI: a brief neuropsychological test battery in Spanish with
norms by age and educational level. J Int Neuropsychol Soc.

[68] Tucker AM, Stern Y (2011). Cognitive reserve in aging. Curr Alzheimer Res.

[69] Lowe PA, Reynolds CR (1999). Age, gender, and education may have little influence on error
patterns in the assessment of set-shifting and rule induction among normal
elderly. Arch Clin Neuropsychol.

[70] Bennett DA, Wilson RS, Schneider JA, Evans DA, Beckett LA, Aggarwal NT (2002). Natural history of mild cognitive impairment in older
persons. Neurology.

[71] Fagundes SD, Silva MT, Thees MFRS, Pereira MG (2011). Systematic review Prevalence of dementia among elderly Brazilian:
a systematic review. Med J.

